# Genome sequences of *Enterobacter asburiae mcr-9* harboring and *Enterobacter roggenkampii mcr-10* harboring isolated from a wastewater treatment plant in Japan

**DOI:** 10.1128/mra.00407-24

**Published:** 2024-09-24

**Authors:** Hiroki Izawa, Ryotaro Eda, Nagi Niida, Masaki Nakamura, Takashi Furukawa, Kazunari Sei, Makoto Kubo, Masato Suzuki, Shotaro Maehana

**Affiliations:** 1 Laboratory of Environmental Microbiology, Graduate School of Medical Sciences, Kitasato University, Kanagawa, Japan; 2 Department of Clinical Laboratory, Kitasato University Hospital, Kanagawa, Japan; 3 Research Center for Biosafety, Laboratory Animal and Pathogen Bank, National Institute of Infectious Diseases, Tokyo, Japan; 4 Department of Laboratory Medicine, School of Medicine, Kitasato University, Kanagawa, Japan; 5 Department of Health Science, Laboratory of Environmental Hygiene, School of Allied Health Sciences, Kitasato University, Kanagawa, Japan; 6 Regenerative Medicine and Cell Design Research Facility, School of Allied Health Sciences, Kitasato University, Kanagawa, Japan; 7 Antimicrobial Resistance Research Center, National Institute of Infectious Diseases, Tokyo, Japan; Department of Biology, Queens College, Queens, New York, USA

**Keywords:** *Enterobacter*, colistin-resistant, *mcr*

## Abstract

Severe infections caused by multi-drug-resistant Gram-negative rods pose a clinical threat due to their high mortality risk. The global spread of plasmid-mediated colistin-resistance genes has become a serious problem. In this study, we identified *Enterobacter* spp. harboring *mcr-9* and *mcr-10* from a wastewater treatment plant in Japan.

## ANNOUNCEMENT

Severe infections caused by multidrug-resistant Gram-negative rods pose a high mortality risk due to limited treatment options. Colistin is the last resort for treating severe infections, but colistin-resistant bacteria, caused by mobile colistin resistance (*mcr*) genes, are a global issue (1). Of the 10 known *mcr* variants (2), *mcr-9* and *mcr-10* are frequently detected in patient (3–5) and environmental samples (6, 7), suggesting their spread. Our analysis of aquatic environments has detected Enterobacterales strains harboring *mcr-9* and *mcr-10*, emphasizing the urgent need for detailed genomic analysis.

*Enterobacter* spp. isolates KAM546 and KAM576 were aerobically grown at 37°C for 18–24 hours on Desoxycholate-Hydrogensulfide-Lactose Agar, and genomic DNA was extracted using Genomic-tips and Genomic DNA Buffer Set (QIAGEN). Whole-genome sequencing was performed using HiSeq X (Illumina) with HiSeq X Ten Reagent Kit v2.5 (300 cycles) and MinION (Oxford Nanopore Technologies: ONT) with the R9.4.1 flow cell. The library for Illumina (paired-end, insert size of 500 to 900  bp) was prepared using the Nextera XT DNA Library Prep Kit, and quality trimming was performed using Trimmomatic v0.38.1 (8) with the default parameters (minimum mean quality = 20). The library for ONT sequencing was prepared with unsheared and non-size-selected DNA FDHL using the Rapid Barcoding Kit (SQK-RBK004) and quality trimming using Filtlong v0.2.1 (9) with the customized parameters (minimum length = 1,000 and minimum mean quality = 10). ONT reads were basecalled using Guppy v5.0.11 with the super-accuracy mode and were assembled *de novo* using Canu v2.1.1 (10) with the default parameters. The overlap regions in the assembled contigs were detected using LAST (https://gitlab.com/mcfrith/last) and then trimmed manually. Sequencing errors were corrected by Racon v1.5.0 (11) twice with the default parameters using MinION reads and then corrected by Pilon v1.20.1 (12) twice with the default parameters using Illumina reads, resulting in their complete genome sequences. The quality of the genome assembly was assessed by CheckM v1.1.3 (13) with default parameters, and genome completeness and contamination were estimated at 99.1% and 0.6% for KAM546, and 99.6% and 0.5% for KAM576, respectively. The complete genome sequences of KAM546 comprised one chromosome and 12 plasmids (accession nos. AP026873 to AP026885), totaling 4,758,695 bp with an average sequencing depth of 44.2×. Similarly, KAM576’s genome consisted of one chromosome and six plasmids (accession nos. AP026886 to AP026892), totaling 5,112,230 bp with a sequencing depth of 41.0× (Table 1). DFAST server (14, 15) annotated 4,639 coding DNA sequences for KAM546 and 4,892 for KAM576 using default parameters.

**TABLE 1:** Genome features of the *Enterobacter* sp. strains

Strains	KAM546	KAM576
Size (bp)	4,758,695	5,112,230
Average sequencing depth	44.2×	41.0×
GC content (%)	55.6	55.6
Coding DNA sequences	4,639	4,892
Genome completeness (%)	99.1	99.6
Contamination (%)	0.6	0.5
ONT Nos. of raw reads	98,371	55,376
ONT N50	7849	8402
Illumina Nos. of raw reads	7.727820 M	10.879164 M
Illumina accession nos.	DRX477820	DRX477821
ONT accession nos.	DRX477822	DRX477823
Closest taxonomic assignation -accession (ANI value to closest species [%])	*Enterobacter roggenkampii* -DSM 16690 (98.46)	*Enterobacter asburiae* -GCA_016027695.1 (96.86)
Closest taxonomic assignation -accession (dDDH value to closest species [d4, in%])	*Enterobacter roggenkampii* -DSM 16690 (87.6)	*Enterobacter asburiae* -AX109549 (74.2)

Using Genome-to-Genome Distance Calculator v2.1 (16) and FastANI v1.3 (17), we identified KAM546 as Enterobacter roggenkampii and KAM576 as Enterobacter asburiae. KAM546 harbored *mcr-9,* and KAM576 harbored *mcr-10* on their chromosomes, along with multidrug resistance transporter genes mdf(A) and oqxAB (18, 19), suggesting that they might have acquired resistance to multiple clinically important antimicrobials.

## Data Availability

Complete genome sequences consisting of chromosomes and plasmids of *E. roggenkampii* KAM546 and *E. asburiae* KAM576 have been deposited at GenBank/EMBL/DDBJ under accession nos. from AP026873 to AP026885 for KAM546 and from AP026886 to AP026892 for KAM576. The version described in this paper is the first version, from AP026873.1 to AP026885.1 and from AP026886.1 to AP026892.1, respectively. The raw sequence data is available in the Sequence Read Archive under the accession nos. from DRX477820 to DRX477823.
